# Dynamics of Ceramide Channels Detected Using a Microfluidic System

**DOI:** 10.1371/journal.pone.0043513

**Published:** 2012-09-12

**Authors:** Chenren Shao, Bing Sun, Don L. DeVoe, Marco Colombini

**Affiliations:** 1 Department of Biology, University of Maryland, College Park, Maryland, United States of America; 2 Department of Mechanical Engineering, University of Maryland, College Park, Maryland, United States of America; J. Heyrovsky Institute of Physical Chemistry, Czech Republic

## Abstract

Ceramide, a proapoptotic sphingolipid, has been shown to form channels, in mitochondrial outer membranes, large enough to translocate proteins. In phospholipid membranes, electrophysiological studies and electron microscopic visualization both report that these channels form in a range of sizes with a modal value of 10 nm in diameter. A hydrogen bonded barrel-like structure consisting of hundreds of ceramide molecules has been proposed for the structure of the channel and this is supported by electrophysiological studies and molecular dynamic simulations. To our knowledge, the mechanical strength and deformability of such a large diameter but extremely thin cylindrical structure has never been reported. Here we present evidence for a reversible mechanical distortion of the cylinder following the addition of La^3+^. A microfluidic system was used to repeatedly lower and then restore the conductance by alternatively perfusing La^3+^ and EDTA. Although aspects of the kinetics of conductance drop and recovery are consistent with a disassembly/diffusion/reassembly model, others are inconsistent with the expected time scale of lateral diffusion of disassembled channel fragments in the membrane. The presence of a residual conductance following La^3+^ treatment and the relationship between the residual conductance and the initial conductance were both indicative of a distortion/recovery process in analogy with a pressure-induced distortion of a flexible cylinder.

## Introduction

**Figure 1:**
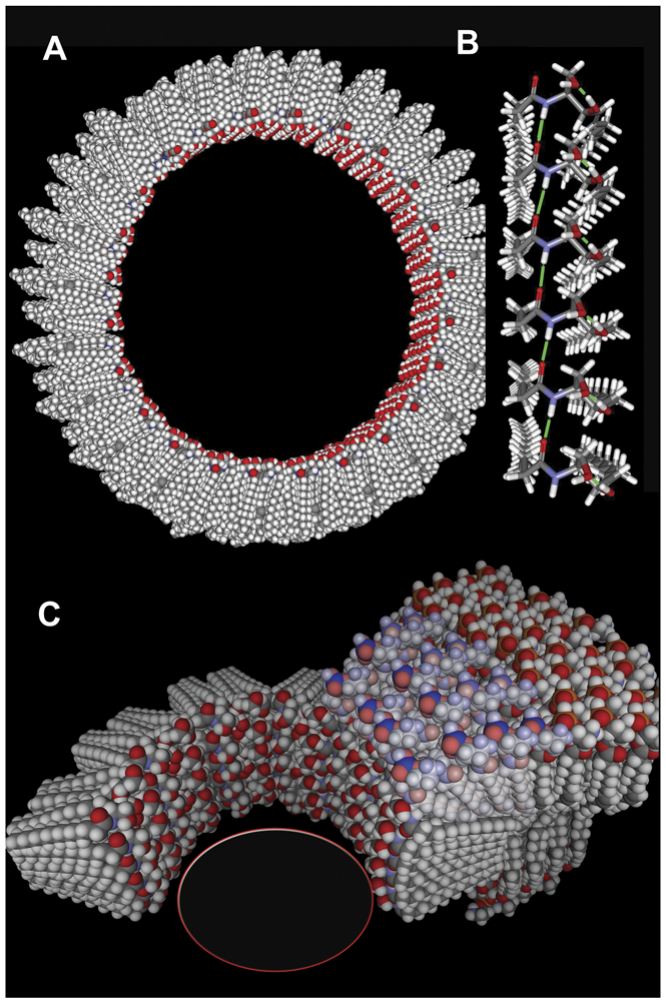
Working model of a ceramide channel. (A) A channel composed of forty-eight columns arranged in an antiparallel fashion. (B) One of these columns is shown is some detail. It is composed of 6 ceramides stacked so as to span the apolar portion of the membrane. Hydrogen bonds (green) between the amide linkages connect adjacent ceramides. The two hydroxyl groups at the polar end of each ceramide also hydrogen bond with neighboring groups. (C) At the interface between the ceramide channel and the phospholipid bilayer some curvature is needed to obtain a continuous polar surface. The interfacial phospholipids must curve to meet the channel and the interfacial ceramides must curve outward resulting in a channel with a somewhat hourglass shape.

The self-assembly of molecules and macromolecules into higher-order structures is commonplace within cells and critical to life as we know it. Common examples are the mitochondrial respiratory complexes, microtubules and microfilaments, ribosomes and membranes. Whereas it is normal and accepted that proteins and RNA form well-organized high-order structures, lipids are relegated to the formation of liquid or solid phases. It is generally assumed that lipids cannot form highly ordered structures save for crystalline or paracrystalline structures. An exception to this has emerged. Ceramide, a sphingolipid, has been shown to form highly-organized channels in phospholipid membranes [1], [2], [3], [4], [5], [6], [7], [8], [9], [10]. Although the dependence of ceramide channel stability on membrane lipid composition is still under investigation, the propensity for the formation of these channels does vary from one natural membrane to another. The erythrocyte plasma membrane is refractory to ceramide channel formation whereas the mitochondrial outer membrane is very sensitive to the formation of these channels [5]. Model-building [8], molecular dynamic simulations [11], and transmission electron microscope (TEM) visualization [10] indicate that these channels are formed by columns of ceramide monomers that span the membrane and assemble to form a barrel-like structure (Fig. 1). In these models, the amide linkage in ceramide forms the hydrogen bonds that connect ceramide monomers into columns, in much the same way that the hydrogen bonds of the amide linkages stabilize the secondary structure of proteins. Adjacent columns are proposed to be connected by the hydrogen bonding of the twin hydroxyls on each ceramide molecule with corresponding residues on adjacent columns. The hydroxyl-hydrogen-bonded network would form the inner lining of the channel, favoring interactions with water and giving the structure mechanical strength. As shown in Fig. 1b, the channel is proposed to interface with the phospholipid bilayer by curvature of both the channel and phospholipid bilayer structure. Thus this higher-order structure requires the particular environment of the phospholipid membrane and the interface with the water phase.

The dynamics of a ceramide channel, a structure composed of hundreds of individual molecules, is largely unexplored because of the difficulty of making structural measurements. The ceramide channels are in dynamic equilibrium with either ceramide monomers or non-conducting ceramide complexes in the membrane. Experimental observations indicate that only about 1% of the ceramide in the membrane is participating in the channel structure. This makes the use of spectroscopic methods to explore the ceramide structure, very difficult. This dynamic equilibrium can be shifted toward channel disassembly by an anti-apoptotic protein called Bcl-x_L_ [6] or by La^3+^ [3]. The latter may be acting by applying lateral pressure to the channel by binding to negatively-charged phospholipids in the membrane. This is how La^3+^ acts to inhibit the function of stretch-sensitive channels [12]. Addition of La^3+^ to ceramide channels formed in planar phospholipid membranes results in disassembly of the channels. This disassembly has features that clearly point to a highly-structured cylindrical barrel-stave channel. The large conductance drops are quantized. They have a strong preference for being multiples of a fundamental unit, 4.0 nS, in 1.0 M KCl [7]. Theoretical calculations show that 4.0 nS is consistent with the loss of a pair of ceramide columns in the working model of the channel. Thus multiples of 4.0 nS indicate disassembly by the preferential loss of an even number of ceramide columns [7]. This is consistent with columns arranged in an anti-parallel fashion, each pair stabilized to some extent by dipole-dipole interactions. The dipoles would come from the alignment of the amide linkages in the columns, similar to the origin of the dipole in the alpha helix. The pattern of large conductance decrements could only be modeled by assuming a rigid cylindrical structure. If the structure was assumed to be flaccid or non-cylindrical the calculations did not produce histograms of conductance changes comparable to the experimental data. La^3+^-induced disassembly occurred at sub-micromolar levels of free La^3+^ and resulted in total loss of conductance.

These previous studies of the structure and dynamics of ceramide channels were performed on planar phospholipid membranes made from monolayers without the use of a liquid hydrocarbon solvent. Current studies use phospholipid membranes made in such a way as to produce a planar membrane attached to the plastic partition by a hexadecane-containing annulus. This annulus is both a source and sink of phospholipids. In this system, La^3+^ additions result in a very different outcome. The ceramide channel remains but its conducting properties change drastically. This is a different type of stress-induced dynamics.

Whereas transient, unstable “lipidic pores” have been described in various contexts [13], [14], ceramide forms large stable channels in phospholipid membranes. This property is, to date, unique for cellular lipids. Ceramide channels form pores that vary greatly in size with a modal value of 10 nm in diameter. The channels are believed to be in dynamic equilibrium with ceramide monomers and ceramide aggregates in the membrane. The channel size observed is presumably the result of this equilibrium and different local conditions result in different sizes. The mechanical stability of a cylindrical structure with such a small aspect ratio (height/diameter) composed of small units that are non-covalently bound and indeed under dynamic equilibrium with non-channel structures, is an unexplored area. Beyond assembly and disassembly, the dynamics of such structures is totally unknown. Here we provide an initial glimpse into what we conclude to be the distortion of the cylindrical channel and the accompanying recoil/relaxation as the original structure is restored.

## Results and Discussion

**Figure 2:**
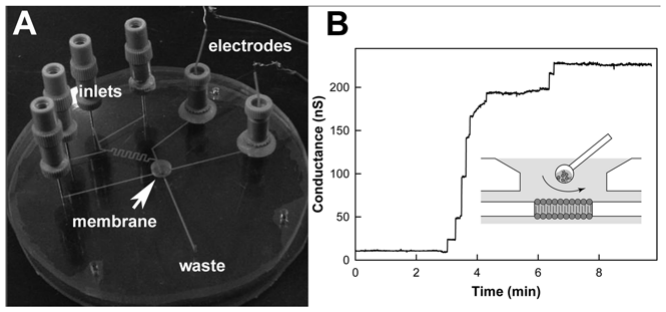
The microfluidic apparatus and a sample recording of channel formation. (A) A fabricated BLM microfluidic bilayer formation “chip” with one buffer inlet, three perfusion inlets, and two Ag/AgCl electrodes. (B) Membrane conductance trace showing the formation of a ceramide channel. The BLM was formed by diffusive painting (inset) as previously described [15], [19].

A microfluidic planar membrane system [15] was used to study the dynamics of ceramide channels by recording changes in conductance following the exposure of the channels to La^3+^ and the removal of the La^3+^ by EDTA treatment. The membrane-forming solution contained C_16_-ceramide at a mole fraction of 1∶50 ceramide to phospholipid. The aqueous solutions contained 0.25 M KCl, 1 mM MgCl_2_, and 20 mM PIPES (pH 6.9). Ceramide channels formed spontaneously in the membrane following the thinning process. An example of channel formation is shown in Fig. 2b. Previous studies have shown that ceramide forms one large single channel in a planar phospholipid membrane formed from monolayers [3]. The results obtained with the microfluidic system are interpreted in this light and thus the step-wise increments in current shown in Fig. 2b are interpreted as incremental steps in the growth of the single channel. As reported previously [3], the sizes of the step-wise conductance increments vary in size and do not show any pattern beyond the expected decrease in frequency with increase in step size. They are perhaps due to pre-form ceramide aggregated combining with the channel, increasing its size by an amount, related to the size of the combining aggregate. An alternative interpretation is that the increments each represent individual channels whose size varies greatly. The latter interpretation would have difficulty explaining published results [3] and some of the results described below.

### La^3+^ addition results in a reduction of membrane conductance

**Figure 3:**
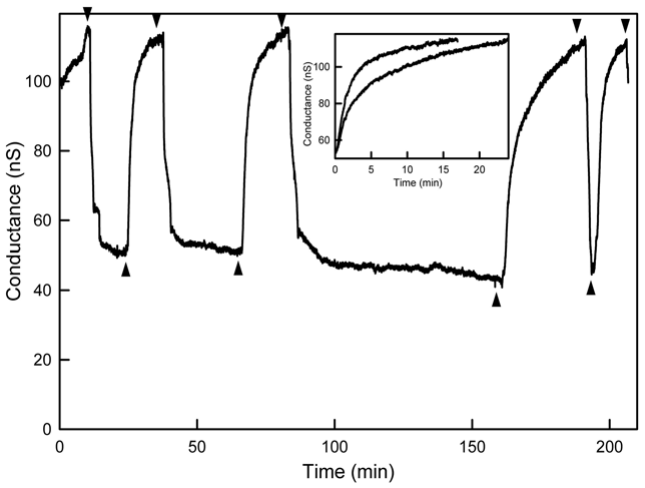
Cyclic changes in ceramide channel conductance following alternative perfusions with La^3+^ (50 µM) and EDTA (50 µM)-containing medium. Downward arrowheads indicate the start of perfusion with La^3+^ medium whereas upward arrowheads indicate the start of perfusion with EDTA-containing medium. The inset shows the overlap of conductance increases following EDTA treatment for short (upper curve) and long (lower curve) pretreatments with La^3+^. The short treatment was the second La^3+^ treatment in the record and the long was the third treatment.

The perfusion of the solution on the lower side of the planar membrane with the same solution but supplemented with 50 µM LaCl_3_ resulted in a drop in conductance as previously described [3] but with a critical difference. The conductance drop only resulted in a partial loss of conductance in most experiments (e.g. Fig. 3). Previous results with membranes made from monolayers invariably resulted in a total loss of conductance. The partial conductance drop observed with the microfluidic system could be due to partial disassembly of the channel or channels resulting from a La^3+^-induced shift in the dynamic equilibrium between ceramide in channels and ceramide monomers or non-conducting structures in the membrane. The increase in lateral pressure induced by this lanthanide [12] could be responsible for altering the dynamic equilibrium by favoring structures with a reduced occupied membrane area. The 2-dimensional pressure in the membrane times the area occupied by a ceramide structure is part of the energy of the structure. Reducing the area by disassembling into structures without an aqueous pore would reduce the energy and thus favor the loss of conductance. The virtual insolubility of ceramide in the aqueous phase excludes the possibility that ceramide leaves the membrane thus ceramide molecules leaving the channel must remain in the membrane in other forms. If this interpretation were correct one might expect that increasing levels of La^3+^ would shift the dynamic equilibrium further toward channel disassembly. However, the use of La^3+^ levels that were 10 times greater did not result in detectably different extents of conductance drop, indicating that some sort of maximal effect had been achieved. The removal of the La^3+^ by perfusion with EDTA generally restored the original conductance level, somewhat surprising for an assembly/disassembly mechanism. As is evident from Fig. 3, this change in conductance can be repeated many times by sequential perfusion with EDTA and La^3+^. The results appear consistent with a two-state structural model.

### Evidence for a ceramide channel disassembly and reassembly model

In contrast to the traditional planar membrane system where turbulent mixing is used to change solute concentrations in large aqueous compartments, with the laminar flow dominated flow in the microchip, changes in the local concentration at the membrane as a function of time can be precisely predicted by using a mathematic model [15]. Using a flow rate of 10 µL/min, the concentration of perfused substance next to the membrane reaches 95% of the final value in 3.6 s. Thus, compared to the rates of change in conductance observed in Fig. 3, the concentration change of La^3+^ and EDTA can be taken as step functions. Thus the time dependent measurements of channel conductance effectively reflect the dynamics of the channel.

**Figure 4:**
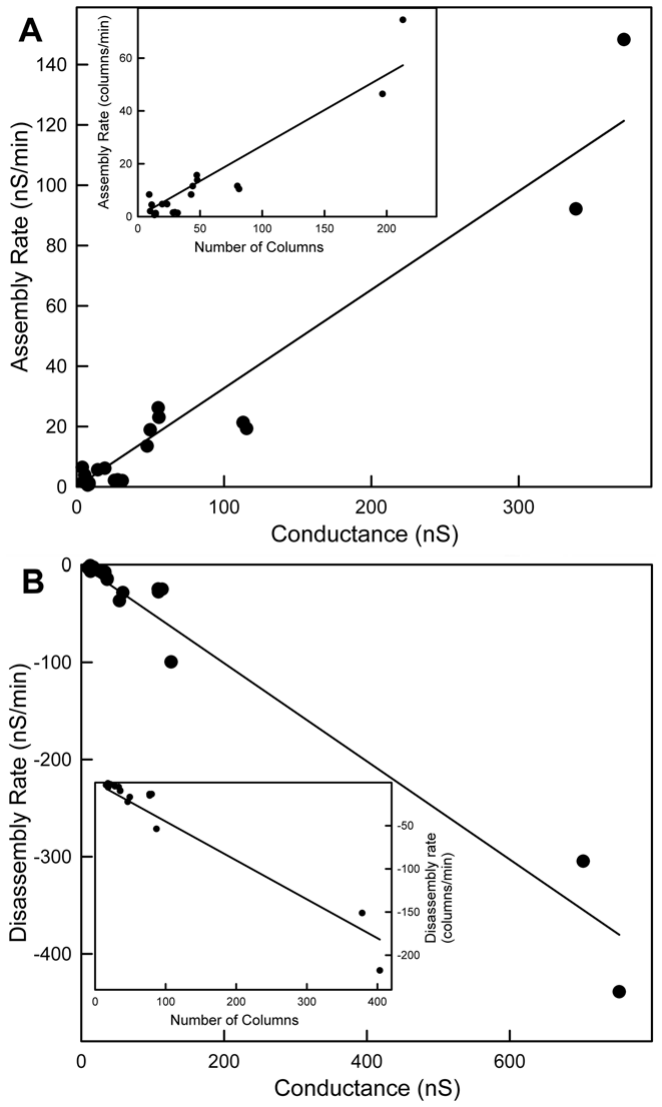
Correlations between the rates of conductance increases and decreases with channel size. The initial rate of conductance decrease (nS/min) is proportional to the starting conductance (nS) (A) (r = 0.96) and the calculated initial rate of column loss (columns/min) is proportional to the starting circumference of columns (inset in (A) (r = 0.94)). The initial rate of conductance increase (nS/min) is proportional to the conductance (nS) just before EDTA perfusion (B) (r = 0.98), and the calculated initial rate of column reassembly (columns/min) is proportional to the circumference of columns before EDTA perfusion (inset in (B) (r = 0.96)).

The initial rates of conductance change were determined by using a linear fit to the initial change in conductance. Both the rates of conductance decrease following exposure to La^3+^ and increase with EDTA perfusion correlated linearly with the conductance before delivery of either La^3+^ or EDTA (Fig. 4). This is consistent with a greater conductance translating into a greater reaction surface. Whether considering one or many channels, a greater conductance would be consistent with more sites at which ceramide columns could leave the channel or insert into the channel. Indeed, considering the formation of a single channel, from the initial conductance the circumference of a cylindrical channel was calculated and the rate of conductance change correlated linearly with the calculated circumference. Thus the results would be consistent with the conclusion that La^3+^ favors channel disassembly and EDTA favors channel growth (by removing La^3+^).

**Figure 5:**
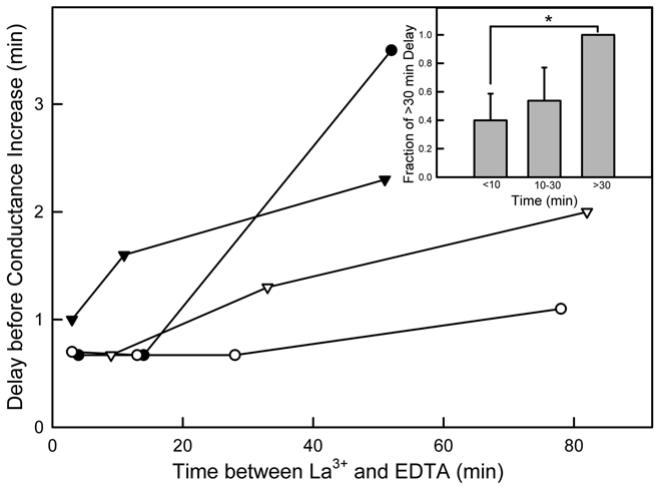
The delay between the delivery of EDTA and the initiation of conductance increase correlates with the time of exposure to La^3+^ prior to EDTA treatment. Each curve is from an independent experiment. Inset: For each experiment, the delay times between the start of EDTA perfusion and the start of conductance increase were grouped as follows: <10 min, 10–30 min, and >30 min based on the length of time of LaCl_3_ treatment and then normalized to the result of the “>30 min” group. The averages ± SD of the relative delay times of the different experiments in each group is shown. The “*” indicates that the “<10 min” group was significantly different from the “>30 min” group at the 95% confidence level.

By varying the time interval between La^3+^ and EDTA perfusions one observes changes in the kinetics of conductance increase. If conductance increase represents channel growth, the changes in kinetics indicate a reduced rate of channel growth with time even though the final conductance achieved remained unchanged (Fig. 3, inset). Previous findings showed that ceramide channel disassembly involved conductance drops consistent with the loss of multiple ceramide columns with preference for the loss of an even number of columns. Thus channel disassembly could result in the formation of structures that reassemble in a channel more easily than the typical ceramide assemblies that may normally exist in the membrane. With time such assemblies could break down or diffuse away resulting in slower rates of channel reassembly. This interpretation fits with the observation of a delay between EDTA perfusion and conductance increase (Fig. 5) and this delay increasing with increasing time between La^3+^ treatment and EDTA treatment. The results from the four experiments were pooled and sorted into three bins (Fig. 5, inset). By doing so a statistically significant difference was obtained between intervals shorter that 10 min and those longer than 30 min.

**Figure 6:**
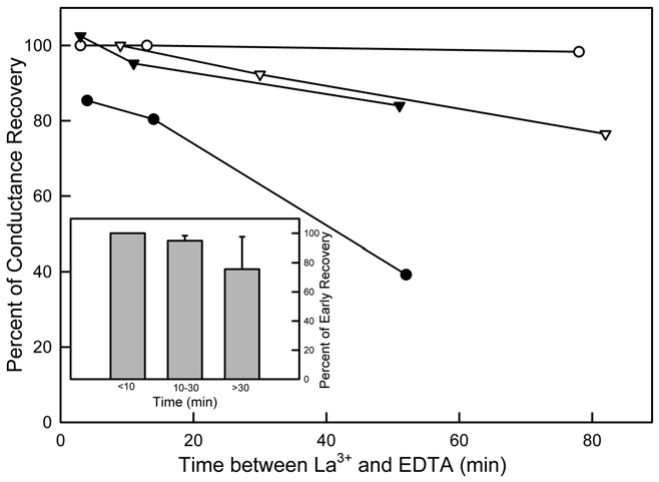
Lack of statistically significant correlation between the percentage of conductance recovery following EDTA treatment and the prior time of exposure to LaCl_3_. Each curve is an independent experiment. The averages of the relative percentage of conductance recovery of different experiments in each group are shown in the inset. The results were grouped and normalized to the values of the “<10 min” group. No statistically significant difference was observed.

While qualitatively consistent with the channel assembly/disassembly hypothesis, the length of time over which the kinetics changed is far too long for a diffusion model. Any fragments that came off the channel would diffuse away rapidly and be unavailable for reassembly during the time at which the conductance increased. Considering the mole fraction of ceramide present in the membrane, there is vastly more ceramide available for channel growth from ceramide in the membrane than any ceramide from a previous putative disassembly event and therefore the changes in kinetics are not easily explained by a disassembly/reassembly model. Furthermore if the channel fragments were to have a special structure allowing preferential reassembly one might expect to measure a reduced conductance recovery after EDTA perfusion that became more pronounced with gap time between LaCl_3_ treatment and EDTA delivery. In Fig. 6, each curve represents multiple alternate perfusions with LaCl_3_ followed by EDTA. Although there seems to be a negative trend, there is no statistical correlation with gap time when results are grouped (Fig. 6, inset).

The observation that is most inconsistent with the assembly/disassembly mechanism is the residual conductance after the La^3+^-induced conductance drop. This and the other inconsistencies are best explained by a different dynamic mechanism.

### Evidence for a ceramide channel distortion model

**Figure 7:**
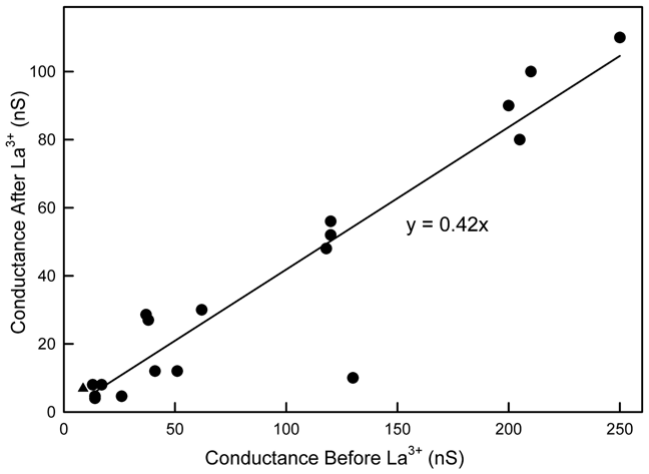
There is a linear relationship between the conductance (nS) of a ceramide channel after LaCl_3_ treatment and the conductance (nS) before LaCl_3_ perfusion (r = 0.94). The circles are results of treatments with 50 µM LaCl_3_ whereas the triangle is an experiment with 500 µM LaCl_3_.

**Figure 8:**
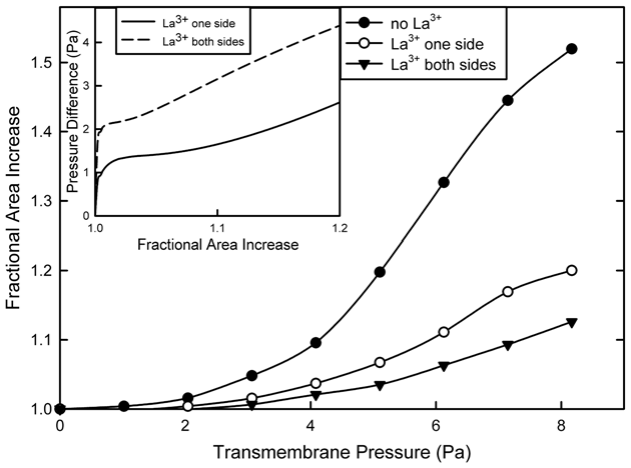
Lanthanum chloride addition increases the transmembrane pressure needed to increase the area of a planar phospholipid membrane. The data of the pressure/area curve was first collected in the absence of LaCl_3_. Then 50 µM LaCl_3_ was added to one side of the same membrane and the data was collected again. Finally 50 µM LaCl_3_ was added also to the other side of the same membrane and the final data set was collected. For the experiments illustrated, the La^3+^ buffer was used (see Methods). Pressure was applied by increasing the level of the solution on one side of the membrane. Inset: Empirical equations were fit to the data in each curve in the main figure. Using these expressions, the difference in pressure needed to achieve the same membrane area with and without LaCl_3_, was calculated. As the subtraction involved the difference between two empirical equations, the full function was plotted in the inset. The results shown are typical of two independent experiments.

An alternative to the assembly/disassembly model is the possibility that a large cylindrical channel could be distorted by the addition of La^3+^, resulting in a reduced cross-sectional area and thus a reduced conductance. La^3+^ removal would allow the channel to relax to its previous state. This would easily explain the transitions between two conducting levels (Fig. 3) and how these levels change with changes in initial conductance (Fig. 7). Note that increasing the [La^3+^] by a factor of 10 did not result in a further drop in conductance (triangle in Fig. 7). Lanthanides are known to inhibit the opening of stretch-sensitive channels by changing the transmembrane profile of the lateral pressure [12], [16], [17] resulting in an increase in the lateral pressure in the hydrocarbon region by 45 mN/m. In a “free” membrane, one is able to expand or contract without outside constraints, and the integral of the membrane tension as a function of distance through the membrane should remain zero. However, the experiments described were performed on a membrane connected to a plastic partition by a torus of phospholipid/cholesterol and hydrocarbon. The partitioning of the lipids between the membrane and the hydrocarbon can be influenced by La^3+^. We performed experiments to test this using the same lipid mixture and hexadecane solvent in the torus. We present these results in Fig. 8. Note that, in the presence of 50 µM La^3+^, a higher transmembrane pressure was needed to increase the surface area of the membrane by introducing curvature. The effect was greater when La^3+^ was added to both sides of the membrane as compared to just one side. This result is consistent with a greater lateral pressure in the membrane keeping phospholipids from flowing in from the torus. As the transmembrane pressure was increased, the lateral pressure in the membrane was reduced until a critical value was reached that allowed phospholipids to flow from the torus into the membrane. The force introduced into the membrane by the transmembrane pressure is countered by two processes: the interlipid cohesion and the resistance to bending of the membrane. In the experimental procedure used, one cannot increase the membrane area without decreasing the radius of curvature. Thus at the same area change, the radius of curvature is the same regardless of treatment with La^3+^. Thus, in the inset, the added transmembrane pressure needed to achieve the same area increase in the presence of La^3+^ is plotted as a function of the fractional area increase. For the one-sided La^3+^ addition, for low changes in area, the added pressure needed is fairly constant, independent of area, indicating the portion of the applied forces needed to balance the greater lateral pressure in the membrane induced by La^3+^. At larger changes in area, the required added transmembrane pressure increased, indicating that the force needed to bend the membrane is greater in the presence of La^3+^. A similar result is seen in the case where La^3+^ was added to both sides. There the constant region is quite limited. Nevertheless, it is interesting that this added transmembrane pressure is roughly double for the two-sided La^3+^ addition as compared to the one-sided addition.

**Figure 9:**
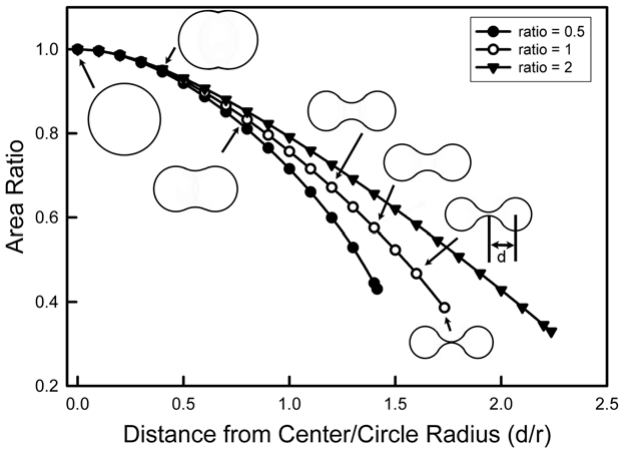
Theoretical calculations of cross-sectional area changes resulting from the formation of a concave structure with positive and negative radii of curvature. The perimeter of the structure was maintained constant as the axial ratio was increased while maintaining the absolute value of the ratio of the two curvatures (negative/positive) at 0.5, 1 and 2 as shown. Calculations were performed at the plotted points. The cross-sections shown are the results of calculations and are to scale relative to each other. The “distance from center referred to on the label of the x-axis is the distance from the center of the structure to the center of the radius of positive curvature, as illustrated.

The increased lateral pressure induced by La^3+^ must favor a structure with a smaller cross-sectional area. The lateral pressure times the area change is the negative energy change that would drive the change in structure. However this change would also result in a change in curvature of the channel wall and this is likely a positive change in energy. For any change to occur the former must be greater than the latter. Experiments show that the structural change occurs slowly and thus it is reasonable to assume that the lateral pressure of the liquid hydrocarbon tails acts uniformly on the channel wall and the tension in the wall counteracts the lateral pressure. Thus the tension in the wall should be uniform at all locations. However, the curvature of the wall cannot be the same at all sections of the wall. Assuming that any stress on the wall would be distributed throughout the structure drastically limits the possible structures. The simplest solution is for the cross-sectional shape of the channel to change from a circle to a biconcave structure. This produces two radii of curvature: one positive and the other negative. The cross-section of such a structure is shown in Fig. 9 at various degrees of distortion. Since the force needed to produce a positive curvature could be different from that required to generate a negative curvature, three plots are shown: one with the radius of negative curvature is half that of the radius of positive curvature (inner curve); the second with both radii having an equal magnitude; the third with the negative curvature being twice that of the positive curvature. In each case, the tension at all parts of the wall of the channel is the same with the ratio of the radii determined by the ratio of the forces needed to generate the two different curvatures. The other constraint is that the circumference is kept constant as the shape changes. With these constraints calculated structures were obtained at increments until the two arcs of negative curvature touched. The area of the biconcave structure as a fraction of the area the original circle is plotted (Fig. 9) against the ratio of the distortion length (d) to the radius of the original circle. As long as the ratio of the two radii of curvature is kept constant, the fractional area change is independent of the size of the original circle (see Appendix S1). This is consistent with the linear dependence of the data shown in Fig. 7. Furthermore, the slope for the conductance ratios of the experimental data, 0.42, is very close to the value of the final area ratio when the two walls with negative curvature touch: 0.39. Similar values were obtained for the other curves. The closeness of the theoretical and experimental values provides confidence that the theory describes the molecular process.

The energetics of the distortion has two counteracting components, the lateral pressure times the area change and the bending of the channel wall times the length of arc associated with the bend. The bending of a structural element, even at the molecular level tends to have a parabolic energy curve (e.g. [18]). Thus the greater the bend the stronger the restoring force, until collapse of some structural element. The results reported with the ceramide channel indicate an elastic restoring force and thus no structural collapse. Thus one would expect that a low-energy minimum might exist, formed by these opposing energy changes. However a comparison of experimental results and theory indicate that the lowest energy is the point at which the two concave surfaces touch, not any point before this event. Supporting this is the observation that regardless of the size of the channel, the ratio of the conductance before and after La^3+^ are the same (Fig. 7). Since the channel is not a continuous material but is composed of staves, the contact between each stave must be the weak point, the hinge that allows the distortion to proceed. With channels of different sizes, the change in the angle between the staves would vary and so would the tension and the restoring force. Thus the fact that the conductance ratio remains constant regardless of channel size, in the size range studied, indicates that the energy change associated with the change in curvature is smaller than the associate energy change due to the decrease in cross-sectional area. Distortion seems to stop when the two negatively curved surfaces make contact, indicating that further distortion results in a structure with a higher energy level.

The changes in kinetics with time considered in light of the assembly/disassembly model can also be understood in the framework of the distortion model. The complexity of the interactions of adjacent ceramide columns would easily accommodate adaptations following the distortion of the structure. These adaptations would reduce the rate of relaxation following the removal of La^3+^. Hysteresis would be expected when an external force is applied to a system made up of hundreds of interacting components.

### Conclusion

Except for channel growth and disassembly, the dynamics of ceramide channels have never before been described. Thanks to the capabilities of the planar membrane microfluidic system developed to study channel dynamics, evidence is presented that ceramide channels can undergo reversible structural changes in response to La^3+^ exposure. The observations are consistent with a partial collapse of a cylindrical channel into a cylindrical structure with a biconcave cross-section. The distortion stores mechanical energy resulting in restoration of the original structure upon chelation of the La^3+^. These findings are consistent with a channel structure that is highly cross-linked by hydrogen bonds and these provide the degree of rigidity and flexibility necessary for both the dynamic motion and the restoring force.

## Materials and Methods

### Reagents

N-hexadecyl-D-*erythro*-sphingosine (C_16_-ceramide), asolectin (soybean polar extract consisting of 45.7% phosphatidylcholine, 22.1% phosphatidylethanolamine, 18.4% phosphatidylinositol and 6.9% phosphatidic acid), 1,2-diphytanoyl-sn-glycero-3-phosphocholine (DPhyPC), and cholesterol were purchased from Avanti Polar Lipids (Alabaster, AL). Ethylenediaminetetraacetic acid (EDTA), ethylenediamine-N,N′-diacetic acid (EDDA), piperazine-N,N′-bis(2-ethanesulfonic acid) (PIPES), 1-hexanol, and n-hexadecane were purchased from Sigma-Aldrich (St. Louis, MO). All other chemicals were reagent grade. Poly(methyl methacrylate) (PMMA) sheets and polyvinylidene chloride (PVDC) film were procured from US Plastics (Lima, Ohio) and Sheffield Plastics (Sheffield, MA), respectively.

### Microfluidic system

The microfluidic bilayer lipid membrane (BLM) chips were fabricated following a protocol described elsewhere [15]. Key components of the perfusion chip include a polyvinylidene chloride (PVDC) film containing an aperture with 80∼150 µm diameter (formed using a hot needle), an open well for painting a film across the aperture, a directly milled bottom channel network (460 µm wide and 150 µm deep) connecting multiple perfusion inlets, and two Ag/AgCl electrodes sealed to the chip by adhesive wax (Fig. 2a). The top and bottom polycarbonate (PC) wafers containing microchannels are thermally bonded on either side of the PVDC film. With three perfusion inlets and a mixer, flow rates of two reagents and one buffer solution can be tuned to achieve different concentrations for each of the chemicals at the lipid membrane.

### Electrophysiological recordings

Planar membranes were formed by a diffusion painting method [15], [19] across a ∼100-µm-diameter hole in the PVDC partition using a solution of 5 mg/mL DPhyPC, 5 mg/mL asolectin, 0.134 mg/mL C_16_-ceramide (1∶50 molar ratio with respect to the phospholipids) and 0.5 mg/mL cholesterol in 10∶1 (v/v) hexanol:hexadecane mixture. The asolectin composition resembles that of mitochondrial lipids and has been used extensively to achieve high activity of mitochondrial complexes (e.g. [20]). The cholesterol is added to mimic the cholesterol in the mitochondrial outer membrane. Ceramide has a higher propensity to forms channels in this lipid mixture as compared to pure lipid membranes (unpublished observation). The DPhyPC was added to increase membrane stability. The lipid composition was not further optimized. Upon membrane formation, the hexanol readily dissolves in the aqueous phase leaving just hexadecane as a solvent. Perfusion flushes away the hexanol. The resulting membrane is supported by a solvent-rich torus which enhances the mechanical resistance of membrane to transmembrane pressure by serving as a source and sink for phospholipids thus allowing for changes in membrane surface area in response to changes in transmembrane pressure. Ceramides self-assemble to form channels. A typical transmembrane conductance trace of ceramide channel formation is shown in Fig. 2b. The aqueous buffer contained 0.25 M KCl, 1 mM MgCl_2_, and 20 mM PIPES (pH 6.9). La^3+^ and EDTA solutions contained 50 µM LaCl_3_ and 50 µM Na_2_EDTA respectively (unless otherwise stated). The Ag/AgCl electrodes were connected to computer through a custom headstage, a 60 Hz noise eliminator (Hum Bug; AutoMate Scientific, CA), filter (LPF-202A, fc = 10 kHz; Warner Instruments, CT) and digitizer (Digidata 1322A; Axon, CA). Clampex 9 software (Molecular Devices, CA) was used to record input voltage and output current data at a 50 kHz sampling rate. Syringe pumps (PHD 2000; Harvard Apparatus, MA) were used to deliver fluids to the needle inlets for perfusion test. The chips and headstage were positioned within a Faraday cage and located on a vibration isolation table to minimize external noise. Clampfit 9 software (Molecular Devices, CA) were used to analyze the collected data.

The membrane stretching experiments were performed with a Lucite chamber consisting of two hemichambers separated by a PVDC partition containing a circular hole 0.43 mm in diameter. Each hemichamber contained 2.5 mL of the aqueous buffer specified above. A membrane was pained across the hole using a the same lipid solution as above except that it did not contain either hexanol or ceramide. The hexanol is washed away in the microfluidic system whereas this chamber was not perfused. Ceramide was not used because the intent was to assess the influence of La^3+^ on the properties of the phospholipid/cholesterol membrane. Transmembrane pressure was applied by addition of aqueous solution to one side of the membrane resulting in a hydrostatic pressure gradient. Changes in membrane area were calculated from capacitance measurements obtained by using a triangular voltage wave (±12 mV, 29.4 Hz) and taking the unit membrane capacitance as 0.8 µF/cm^2^ [21]. After each change in pressure the membrane was allowed to relax to its new condition prior to collecting capacitance measurements. Measurements with and without 50 µM La^3+^ were made on the same membrane. La^3+^ was either added as LaCl_3_ (12.5 µL of 10 mM LaCl_3_) or as a La^3+^ buffer. When the La^3+^ buffer was used, the solution was supplemented with 6.00 mM ethylenediamine-N,N′-diacetic acid (EDDA) prior to membrane formation. At the point of La^3+^ addition, 25 µL of 213 mM LaCl_3_ and 20 µL of 400 mM KOH were added (the KOH consumed the protons released by La^3+^ binding to EDDA maintaining pH 6.9). The design of the La^3+^ buffer and binding constants were published previously [3]. The La^3+^ buffer compensated for loss of La^3+^ from binding to surfaces and was expected to be a better mimic of the perfusion system. However, essentially the same results were obtained by either method.

### Calculations and statistics

Circumferences of ceramide channels were calculated assuming a cylinder 5 nm in length and with a circular cross-section and an internal conductivity equal to that of the bulk phase. The access resistance was included in the calculations as follows:
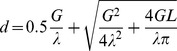
where d is the diameter of the channel; G is the conductance; λ is the conductivity; L is the length of the channel (5 nm). All statistical tests were either the Student's T test or the correlation coefficient (r).

## Supporting Information

Appendix S1 (DOC)
