# Supplementary material for: Dynamics of Ceramide Channels Detected Using a Microfluidic System
Source: PLoS One. 2012 Sep 12;7(9):e43513. doi: 10.1371/journal.pone.0043513 (PMC3440423; doi:10.1371/journal.pone.0043513)
Supplement: Appendix S1 — (DOC) [file pone.0043513.s001.doc]

# Dynamics of Ceramide Channels Detected Using a Microfluidic System

Chenren Shao^†^, Bing Sun^‡^, Don L. DeVoe^†^, Marco Colombini^‡,*^

## Supplemental File

## Appendix

##
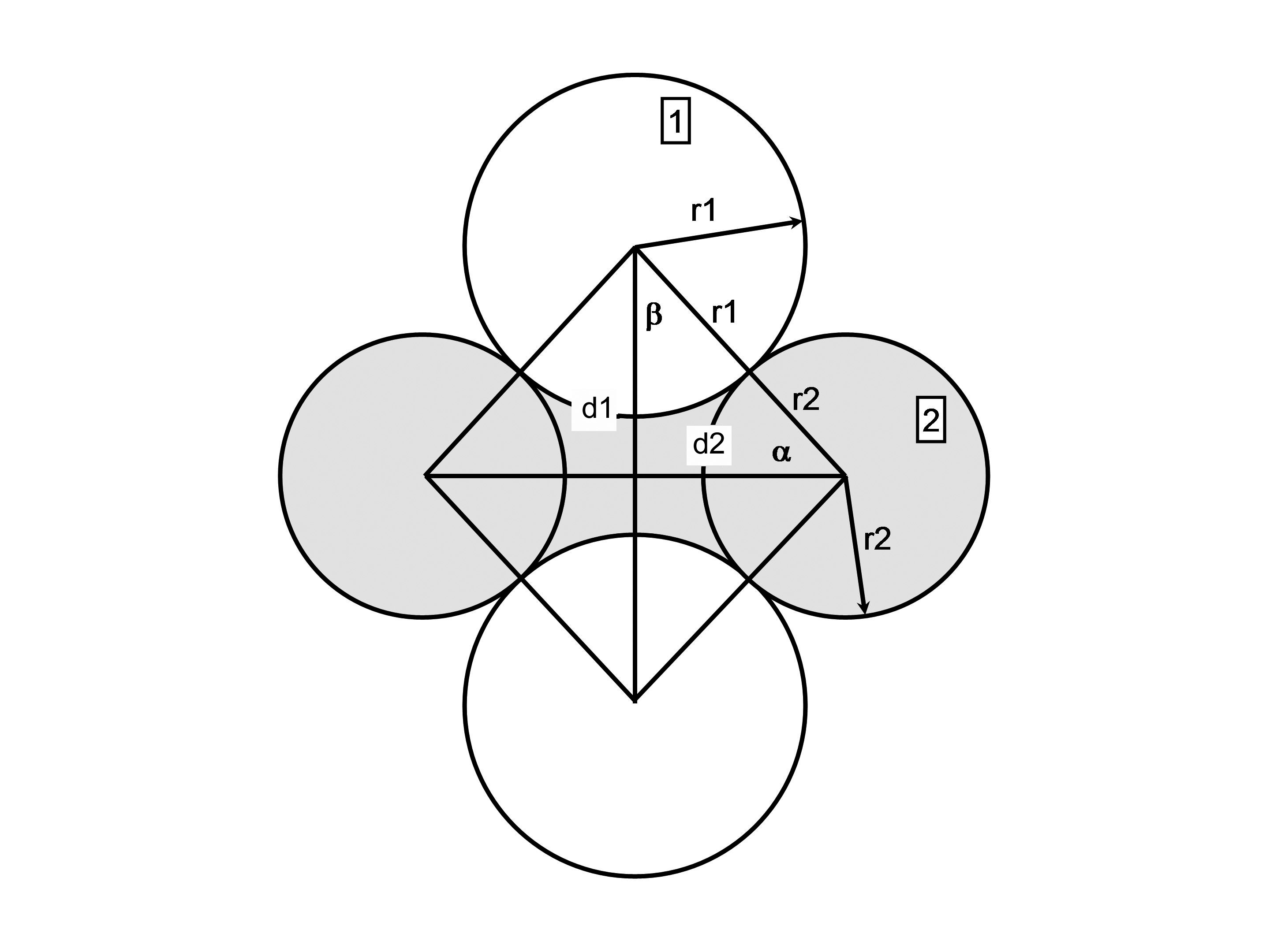


One of the biconcave structures is shown in grey along with the geometrical tools needed to quantitate the relevant parameters. Circles 1 and 2 define the radii of curvature of the negatively and positively curved regions resp. The ratio of the radii (r_1_ and r_2_) of the circles reflect the resistance to bending of the wall of the channel in the convex and concave direction (from inside the biconcave structure). α and β are the angles. d_1_ and d_2_ are the distances from the center of the drawing to the center of circles 1 and 2.

The circumference of the biconcave structure $=4r_{1}\beta+4r_{2}(\pi-\alpha)$

The area of the biconcave structure $=2d_{1}d_{2}+2r_{2}^{2}(\pi-\alpha)-2r_{1}^{2}\beta$

As the distortion of the circle into the biconcave structure was performed, the circumference was kept constant.

Let $\frac{r_{1}}{r_{2}}=x$

Then the area of the biconcave structure = $r_{2}^{2}\left[(x+1)^{2}\sin(2\alpha)+\pi+2\beta-2x^{2}\beta\right]$

The area of the original circle = $r_{2}^{2}\left[\frac{4x^{2}\beta^{2}}{\pi}+\frac{4\beta^{2}}{\pi}+\pi+\frac{8x\beta^{2}}{\pi}+4x\beta+4\beta\right]$

Note that the ratio of the areas is independent of the radii of the circles as long as the ratio of the radii was kept constant.
